# Perfluorodecalin allows resuspension and prevents sediment solidification of extended-release drug formulations in primary packaging

**DOI:** 10.1007/s13346-024-01598-7

**Published:** 2024-05-04

**Authors:** Daniel Primavessy, Sarah Büttner, Sigrid Saaler-Reinhardt

**Affiliations:** 1 Midas Pharma GmbH, Rheinstrasse 49, 55218 Ingelheim am Rhein, Germany; 2 EDUMO Consulting, Professor-Neeb-Strasse 4, 55291 Saulheim, Germany; 3 Institute of Organismic and Molecular Evolution, Johannes Gutenberg Universität Mainz, J. J. Becher-Weg 30A, 55128 Mainz, Germany

**Keywords:** Combination products, Injectables, Drug delivery, Biologics, Extended-release, Suspensions

## Abstract

In this technical note we show with two simple experiments how Perfluorodecalin (PFD), an injectable perfluorocarbon, can be used as an agent for resuspending microparticulate suspensions in primary packaging containers for injection. Furthermore, we explain how this can be a substantial improvement regarding patient compliance in comparison to the commonly used gas headspace for resuspension. Our experiments are conducted with poly(lactic-co-glycolic acid) particles (often used in extended-release pharmaceutical formulations for injection) and in primary packaging that is commonly used in injection devices (glass cartridges). The results show that our method is feasible for resuspension and moreover even sediment solidification/caking is reduced. The differences between the two datasets collected are statistically significant with *p* < 0.01 in both cases.

**Graphical Abstract:**

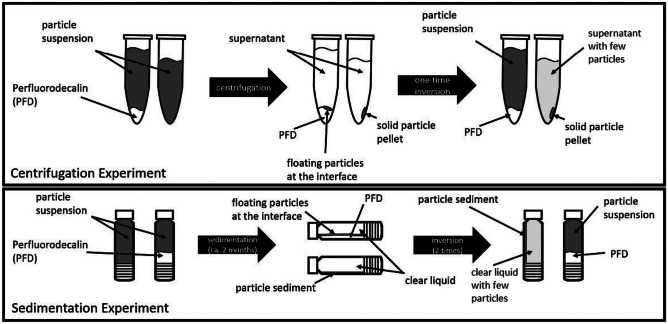

## Introduction

 Among medical devices autoinjectors and injection pumps are one of the hottest topics in the pharmaceutical industry, because they are able to deliver the highly profitable but sensitive biologic therapeutics. Some of these biologics come as injectable extended-release formulations. For example the therapeutic Exenatide, a peptide composed of 39 amino acids comes formulated into poly(lactic-co-glycolic acid) (PLGA) particles in medium-chain triglycerides [1] in an autoinjector (BYDUREON^®^ BCise^®^ from AstraZeneca). The high difference in density between the polymeric particles and the oil in which they are dispersed leads to a quick sedimentation and necessitates the patient to resuspend the suspension strongly prior to the use of the device. In this specific example the autoinjector contains a large gas headspace to enable resuspension but more generally this example illustrates the problem of sedimentation for injectable suspensions in autoinjector devices and pre-filled syringes [2].

Perfluorocarbons like Perfluorodecalin are hydrophobic, oleophobic and chemically inert. That means they are avoiding hydrocarbons as well as polar domains of proteins, which is important to avoid unfolding or agglomeration of biologics on the interface, and do not react with them in any way [3]. Furthermore, PFD has been used in blood volume expanders (e.g., Fluosol [4, 5]), as tamponade for intravitreal application [6, 7] and as contrast medium in echo cardiography [8]. In case of blood-volume expanders, which may be the most impressive application of an emulsion of PFD, poloxamer 188 and perfluorotripropylamine with small amounts of egg yolk and phospholipids were used. The first-generation product Fluosol-DA was developed in Japan and approved in the United States by the Foods and Drugs Administration. Applied amounts as high as 975 mL with 10.6% perfluorocarbon content were used [9]. The 2nd generation product Oxygent™ never made it to approval but experiments in 87 patients with amounts of up to 1.8 g/kg perfluorocarbon content are reported with only minor side effects [10]. Also, a variety of products made from similar perfluorocarbons like octafluoropropane are (or were) on the market as contrast agent for echo cardiography. Both, blood volume expanders and contrast agents, are applied as emulsions as the non-polarity of the fluor stabilizes oxygen bubbles between perfluorocarbon drops. The elimination of PFD from the human body happens on the pulmonary route [11], such that it can be injected along with a drug product. Toxicity data can mainly be found for ophthalmic applications, where it is used as filler for the vitreous body. As large bulk amounts cannot be cleared from the eye it can result in toxicity if not removed manually.

The goal of this work was to demonstrate the use of small amounts of PFD (e.g., 0.5 mL) as a suitable substance for the redispersion of suspensions in primary packaging containers (e.g. ~3 mL). This will provide a basis for further using it in pharmaceutical drug suspensions in injection devices. The amount of PFD was chosen in relation to the amount of particle suspension (6:1), the filling method and in range of a gas headspace reasonable of microparticulate injectable formulations (~ 15%).

## Materials and methods

### Materials

#### Chemicals

Cis-, trans-Perfluorodecalin 95% was ordered from Merck KGaA/Sigma-Aldrich (Darmstadt, Germany). PLGA was obtained as Resomer RG 503 H from Evonik Industries (Darmstadt, Germany). Polyvinyl alcohol (PVA), Type: Mowiol 4–88, was obtained from Kuraray Co., Ltd. (Tokyo, Japan).

#### Primary packaging

As primary packaging 3 mL ISO glass cartridges (siliconized) were obtained pre-crimped from Schott AG (Mainz, Germany). The cartridges were crimped with Bi-Layer combiseals (6194) by West Pharma (Eschweiler, Germany). The plunger stoppers used were West NovaPure WP-457.

Glass cartridges of this type are a common primary packaging of injectables. The glass cartridges were used for the sedimentation experiment, while for the centrifugation experiment common Eppendorf-tube-like 2 mL plastic tubes were used.

#### Machines

For particle preparation we utilized an Ultra-Turrax T25 digital with S25N-G8 tool manufactured by IKA^®^-Werke GmbH & CO. KG (Staufen, Germany). For optical measurements a Tecan Reader infinite i200 was used (Tecan Trading AG, Switzerland). For particle size measurements a Zetasizer Ultra by Malvern Panalytical GmbH (Kassel, Germany) was employed.

### Methods

#### Statistical analysis

A custom-made python script was used to calculate statistical tests. For differing between sample and control sets one-sided, two-sample welch-tests were used (99% confidence). For the outlier a two-sided, one-sample t-test was used (95% confidence). For consecutive tests a Bonferroni correction was applied. Mean values were calculated as arithmetic mean, standard deviations were calculated from unbiased sample variance.

#### Preparation of PLGA particles

PLGA particles were prepared with an emulsion solvent diffusion method: 50 mg of PLGA were dissolved in 1.5 mL of ethyl acetate. Then 2.5 mL of an aqueous PVA solution (2%) were added. The resulting ~ 4 mL were homogenized with the UltraTurrax for 30 s with 10,000 RPM in order to obtain an ethyl acetate in water emulsion. To precipitate the particles from the emulsion 16 mL of the PVA solution were added to the emulsion. This resulted in the precipitation of the particles from the emulsion droplets and the dissolution of the partially miscible ethyl acetate in water. The liquid mixture was left under a hood with constant stirring of ~ 400 RPM for evaporation of the ethyl acetate.

In order to obtain the final particle suspension, the process was conducted 5 times. All 5 suspensions were pooled and after ethyl acetate had evaporated the volume was adjusted to 200 mL with MilliQ water. The resulting PLGA solution was thus 250 mg PLGA particles in 200 mL liquid.

#### Centrifugation experiment

Eppendorf tubes were used for the centrifugation experiment. All tubes (6 samples and 6 controls) were filled with 1.5 mL of particle suspension. The 6 samples were additionally filled with 0.25 mL of PFD. Additionally, 3 tubes were filled with particle suspension to use the supernatant later as background measurement of the optical analysis.

All tubes were centrifuged with a Sigma 3-30KS centrifuge with 30,000 RCF for 30 min at 20 °C.

After centrifugation, the 12 tubes were taken from the centrifuge and inverted once for resuspension. 1 mL of the supernatant (after one inversion) was removed from the vial for measurement. For the measurement 200 µL of the removed supernatants were put into a 96-well plate. Furthermore, 200 µL of the 3 background controls were put into the plate. The plate was measured at 430 nm wavelength for absorption. The expectation here is that samples which resuspended better have a higher absorption because more particles float in the liquid.

#### Sedimentation experiment

The cartridges had to be filled nearly air bubble free. Remaining air was only permissible in a quantity where it would not contribute to a resuspension if the cartridge was inverted. In order to achieve this, cartridges were filled to the maximum possible point and a plunger stopper was put on the end of the cartridge while sliding it over the liquid, bulging out of the cartridge, sideways. It was then pressed a small part into the cartridge. Then a cannula was sticked through plunger stopper sideways, and the plunger stopper was pushed into the cartridge allowing the displaced liquid to leave through the cannula. The plunger stopper was inserted into the cartridge until the end of it met the end of the cartridge. The cannula which was now pinched between the glass of the cartridge and the plunger stopper was removed. The total volume inside the cartridge was thus ~ 3.5 mL. The amount of PFD in the samples was chosen to be 0.5 mL. With a relation of 6:1 of the two liquids to each other, the PFD amount was large enough to be more than ‘just a small bubble’ and small enough to be the minor part to the ‘formulation’ and an easy to use total volume.

Six sample cartridges were filled with 0.5 mL PFD and then with water-based particle suspension to the top (~ 3 mL). For the 6 controls the cartridges were filled first with 0.5 mL MilliQ water and then to the top with water-based particle suspension. This way the total amount of particles in both cartridge sets was be the same, which we deemed important when trying to get the experiment as close to conditions of a real medicine where the amount of particles correlates with the dose of the medicine. To compensate this dilution of 1:1.143 in the controls of the sedimentation experiment, all measured absorption values of the controls were multiplied with 1.143. The samples were stored at 2–8 °C in a refrigerator.

After roughly two months of sedimentation (55 days) the cartridges were retrieved from the refrigerator and brought to the laboratory bench (room temperature). They were taken out of the box in which they were stored without being tilted. Then they were inverted twice for resuspension. Following that a needle was pierced through the septum of the cartridge and the cartridge was pointed downwards. Then the plunger stopper at the back of the of the cartridge was pushed with plunger rod taken from a syringe in order to press liquid out through the needle into an Eppendorf tube. (Note: for the samples with PFD the needle was always pushed deep enough into the cartridge to bypass the PFD which would run to the tip of the cartridge because of its high density.) This way approximately 1.5 mL of potentially resuspended particles were withdrawn from the cartridges and put into Eppendorf tubes.

The retrieved liquid was then resuspended to homogeneity and 200 µL of each was pipetted into a well of a 96-well plate. The plate was measured analogously to the centrifugation experiment.

## Results and discussion

### Particle results

Pooled particles were measured with a Malvern Zetasizer Ultra to determine their approximate size. The measurement was taken through the backward scatter optics with water as dispersant. The measured size of the particles was 1271 nm (Z-Average) with a high polydispersity index (0.797). Though high, we considered it to be acceptable as all experiments were conducted with the same particle suspension and the only important requirements for the particles was a size large enough to sediment, which was the case.

### Experiment results

#### Centrifugation experiment

**Fig. 1:**
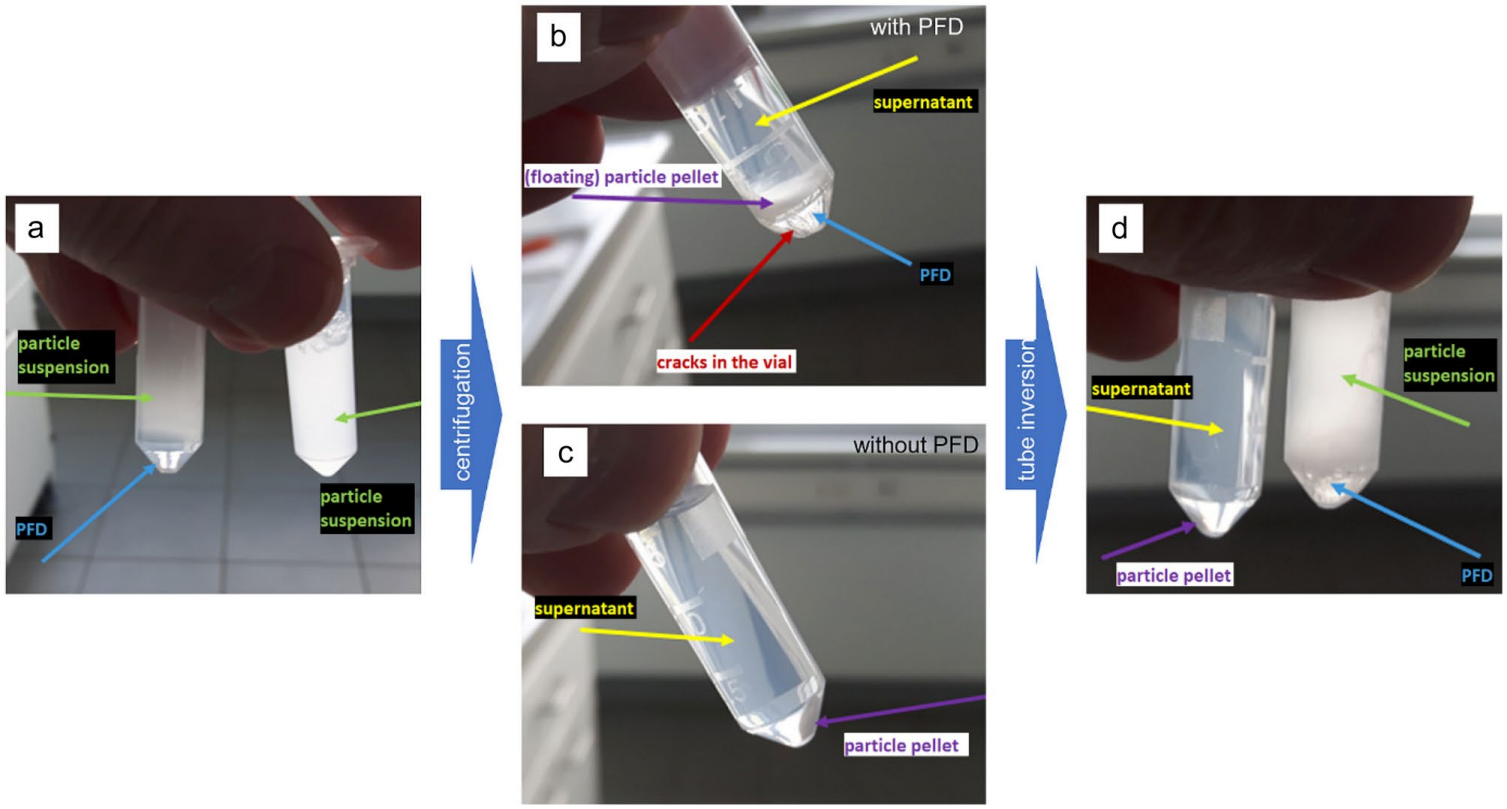
This figure shows the process of the centrifugation experiment in the tubes. Image **a** displays a sample (left) and a control (right) right after filling. Image **b** displays the sample after centrifugation with the particles at the interface. **c** displays the control after centrifugation with the pellet at the bottom of the vial and Image **d** shows both tubes after one-time inversion with the sample on the right and the control on left. Blue arrows indicate PFD, green arrows particle suspension, yellow arrows show supernatant (mostly particle free), purple arrows show pellets and the red arrow points at small cracks in the vial, probably due to the strong centrifugation with dense liquid

The goal of the centrifugation experiment was to demonstrate that PFD prevents sediment solidification. The high force during a centrifugation of 30,000 g for 30 min did not only draw the particles to the bottom of the tube, but also compressed them in a pellet. Since PFD (1.92 g/cm^3^) has a higher density than PLGA (1.53 g/cm^3^), the PLGA particles remain on the interface between the aqueous solution and the PFD. On top of a liquid the solidification does not take place and a simple inversion of the vial can release the pellet. In contrast to the PFD samples the controls had hard pellets centrifuged down on the bottom of the tube. The whole process is illustrated by the images in Fig. 1.

The samples were measured photometrically at 430 nm and the absorption values with mean and standard deviation can be seen in Table 1. The first sample of the controls was a pipetting mistake and therefore not used for further calculations.

**Table 1:** Absorption values of the two experiments

samples with PFD (centrifugation)	#1	#2	#3	#4	#5	#6	MV	SD
	0.842	0.751	0.771	0.738	0.620	0.746	0.745	0.072
controls without PFD (centrifugation)	#1	#2	#3	#4	#5	#6	MV	SD
	0.512	0.331	0.233	0.265	0.303	0.319	0.290	0.040
samples with PFD (sedimentation)	#1	#2	#3	#4	#5	#6	MV	SD
	0.927	0.455	0.604	0.733	0.842	0.687	0.708	0.168
controls without PFD (sedimentation)	#1	#2	#3	#4	#5	#6	MV	SD
	0.014	0.245	0.007	0.016	0.002	0.009	0.009	0.006

Table 1 shows the measured absorption values for samples with and without PFD for the centrifugation and the sedimentation experiment. For all values the mean value of the measured supernatants from the centrifugation experiments was subtracted. This way a possible influence of the 96-well plate, a possible influence of the PVA solution was subtracted. Furthermore, for the controls of the sedimentation experiment the dilution factor was applied. The first value of the controls without PFD for the centrifugation experiment was a preparation mistake, the second value of the controls of the sedimentation mistake is statistically significant outlier. Both values were therefore not considered in the MV and SD calculation.

**Fig. 2:**
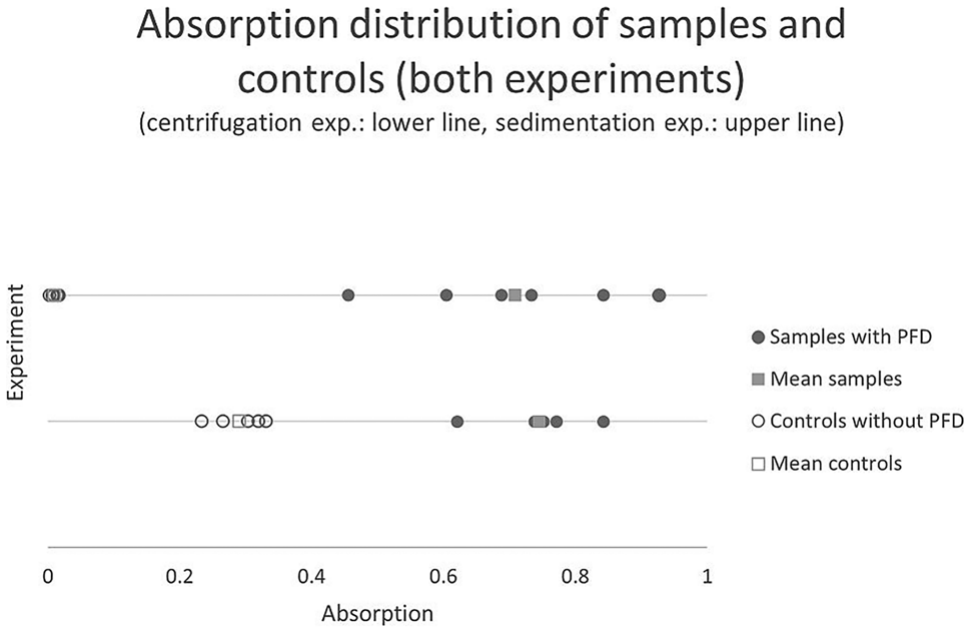
The graph shows the absorption values of both experiments. The sedimentation experiment is shown in the upper line, while the centrifugation experiment is the lower line. The distribution of samples with PFD on the right side as filled symbols (above 0.4) and the distribution of controls without PFD on the left as hollow symbols (below 0.4). The square symbols are the mean values (filled symbols with PFD) and hollow symbols without PFD. In the sedimentation experiment one value of the control was removed because it was statistically proven and outlier. In the centrifugation experiment a control value was removed because there was preparation mistake

The results of the experiment were plotted in Fig. 2 in order display both distributions visually. A t-test (one-sided two-samples Welch test) concluded both distributions to be significantly different from each other at 99% confidence and a p-value of 4.79*10^−7^.

The experiment shows well the difference between both sets and illustrates how PFD can avoid sediment solidification; however, since it is impossible to fill eppendorf tubes without an air bubble it was not possible to demonstrate that a drop of PFD generally is able to resuspend a sediment. To prove this the sedimentation experiment was set up.

#### Sedimentation experiment

**Fig. 3:**
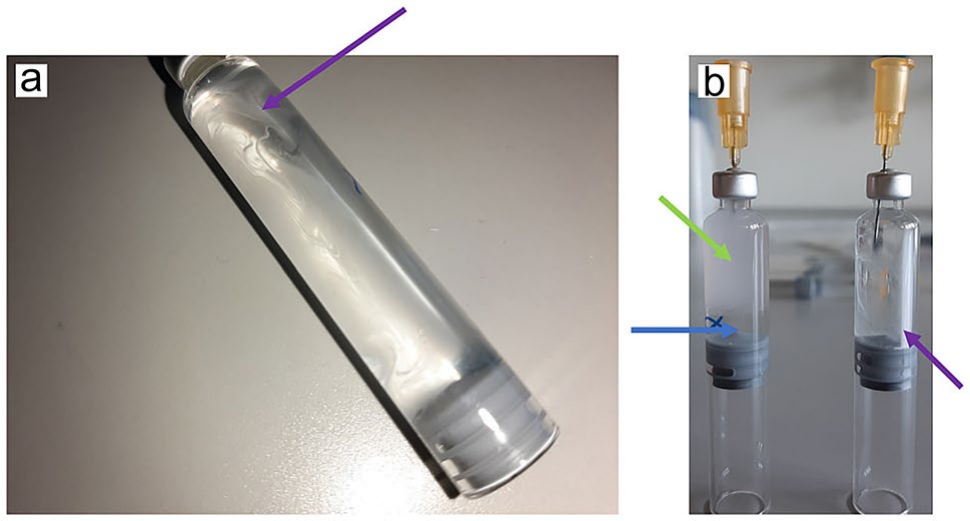
Image **a** shows a cartridge filled with particle suspension and PFD after 55 days of sedimentation at 4 °C. The purple arrow points at some of the streaks which come from particles sedimented onto the PFD interface. Image **b** shows two cartridges after withdrawal of liquid for the measurements. The left one if with PFD (blue arrow). The particle suspension (green arrow) resuspended well, while for the right cartridge the liquid is not well mixed and a white line (the sediment indicated with a purple arrow) is still attached to the glass surface

Particles sedimented well during the 55 days at 4 °C. When the samples were taken from the fridge the liquid was clear and a sediment was observable. While for the controls without PFD a white line of sediment in the lower part of the cartridge could be seen, for the samples with PFD white streaks floating on the PFD could be seen (see Fig. 3a). After inverting cartridges twice for resuspension, samples with PFD were quite well mixed while samples without PFD only showed rudimentary mixing (see Fig. 3b).

To compensate the dilution of 0.5 mL to 3 mL during the preparation of the controls of the sedimentation experiment, all measured absorption values of the controls were multiplied with the dilution factor 1.143. The dilution factors come in place due to the 1:(1+1/7) dilution of 0.5 mL dilution volume on 3.5 mL total volume. Absorption values of controls without PFD were between 0.002 and 0.014 with one exception which was 0.245. This number is so much different that the assumption is justified that it is an outlier, possibly due to a preparation mistake that was not detected. In order to validate this statistically a one-sample t-test (two-sided) was conducted for testing that one possible outlier against the distribution of all other values. At 95% confidence and with a p-value of 3.83*10^−8^ due to statistical significance it is to expect, that it is an outlier due to a different effect. The remaining values have a mean value of 0.009 with a standard deviation of 0.006. The outlier is ~ 16 times larger than MV+SD. It is thus unlikely that the other measured values of the control are affected by the same effect as the outlier. Also we consider 5 values to be sufficiently large data set for the given purpose to continue working with it.

Both sets of values were listed in Table 1 visually plotted in Fig. 2. A t-test was conducted to prove significant difference between both sets. The one-sided, two-samples Welch test with 99% confidence showed the two sample sets to be significantly different with *p* = 7.83*10^−5^. Since value #2 of the controls without PFD was statistically removed from the sample set before, a Bonferroni correction was used for this test.

## Conclusion

One of the biggest problems of extended-release injectable drug formulations is the sedimentation of the particles over time and their necessary homogenous resuspension before injection. In case of self-administration by the patient the drug formulation often comes in injection devices. Patients have to shake the devices sufficiently in order to resuspend the drug suspension prior to use. This can be a hindrance for sick and elderly people as it necessitates the ability of quick arm movement and enough power and may end up in reducing patient compliance. The solution presented in this article provides a substantial improvement as resuspension is much easier due to reduced or no sediment solidification.

The sedimentation experiment shows very well that a resuspension of a sedimented particle suspension is possible with PFD as ‘physical object’ and the centrifugation experiment shows that a solidification (or caking) of the sediment can be avoided/reduced due to the high density of PFD compared to the water-based suspension. Both experiments could be verified statistically.

Furthermore, in common autoinjectors gas headspaces are necessary to allow resuspension but they have to be removed prior to injection which necessitates a complex sequence of preparations. In case of the BYDUREON^®^ BCise^®^ a patient has to point the autoinjector device upwards, unlock and screw it. This sequence primes the device and removes the gas headspace. Only after these steps the patient can inject the substance. Here again is a risk that especially elderly people may misunderstand or forget to follow the instructions correctly. This may result in the removal of drug substance instead of the gas headspace rendering the medicine unusable. The use of PFD would allow suspension drug formulations without gas headspace, making the handling of autoinjectors more convenient for the patients. Important to note is that PFD would not have to be removed in this case as it could be injected into the body along with the medicine.

Further studies will have to determine the exact toxicity of PFD in the subcutaneous and intramuscular space, as it has only been determined for intravenous application yet. Also, from a toxicological point of view a minimization of the amount of PFD in the primary packaging is necessary. As the minimum amount is very much dependent on formulation parameters like viscosity, volume, particle size and density, application frequency, route of application and the used primary container, a minimization will have to be carried out for every potential medicament individually. As for the medicament BYDUREON^®^ BCise^®^, by AstraZeneca, mentioned in the introduction. The used volume is 0.85 mL. In case scalability would be given and we ignore other formulation parameters this would account for 0.142 mL of PFD.

## Data Availability

The datasets generated during and/or analyzed during the current study are not publicly available due to company policy but are available from the corresponding author on reasonable request.

## Abbreviations

MV: Arithmetic mean value
SD: Standard deviation
PFD: Perfluorodecalin
PLGA: poly(lactic-co-glycolic acid)
